# Senescent cancer-associated fibroblasts facilitate tumor associated neutrophil recruitment suppressing tumor immunity

**DOI:** 10.1186/s12967-024-05017-w

**Published:** 2024-03-03

**Authors:** Dongqi Lin, Xiaoqian Zhai, Xinxin Qi, Qinghua Zhou, Yanyang Liu, Yiyun Lin, Jiewei Liu

**Affiliations:** 1 Department of Thoracic Surgery, West China Hospital, Sichuan University, Chengdu, Sichuan China; 2 Department of Medical Oncology, Cancer Center, West China Hospital, Sichuan University, Chengdu, Sichuan China; 3 Lung Cancer Center, West China Hospital, Sichuan University, Chengdu, Sichuan China; 4 Out-Patient Department, The 964Th Hospital of the Joint Logistics Support Force of the Chinese People’s Liberation Army, Changchun, Jilin China; 5 Graduate School of Biomedical Sciences, UT MD Anderson Cancer Center, Houston, TX 77030 USA

**To the editor,**

Anti-PD-1/PD-L1 immunotherapy has demonstrated significant efficacy and promising by activating the body's inherent cytotoxic T cell function [1]. The 5-year OS increased from 11.3% to 19.4% in patients receiving pembrolizumab plus pemetrexed-platinum treatment compared to chemotherapy alone [1]. However, its effectiveness is limited due to the heterogeneity and complexity of the tumor microenvironment, not all patients benefit from this therapeutic approach. Therefore, a comprehensive exploration of the mechanisms underlying immune suppression holds great significance in prognosis for NSCLC patients.

In the proposed third edition of the cancer hallmark in 2022, cellular senescence has been newly recognized as significant hallmarks of tumors. Senescent cells are characterized by their transient cell cycle arrest, pronounced metabolic activity, and anti-apoptotic characteristics. Notably, the metabolic activity of senescent cells represents their secretion of an array of cytokines, chemokines, growth factors, secreted proteases, and insoluble proteins collectively, name as the senescence-associated secretory phenotype (SASP). SASP has been implicated in inducing inflammation and contributing to tumor immunosuppression, thereby promoting tumor progression [2]. In our study, we found senescent cells increased within NSCLC tissues, predominantly comprising of senescent cancer-associated fibroblasts (CAFs). Consequently, this study focuses on exploring the impact of CAFs senescence on tumor immunity in lung adenocarcinoma (Additional file 1).

## Result

### The proportion of senescence cell in lung adenocarcinoma tissues increased

**Fig. 1:**
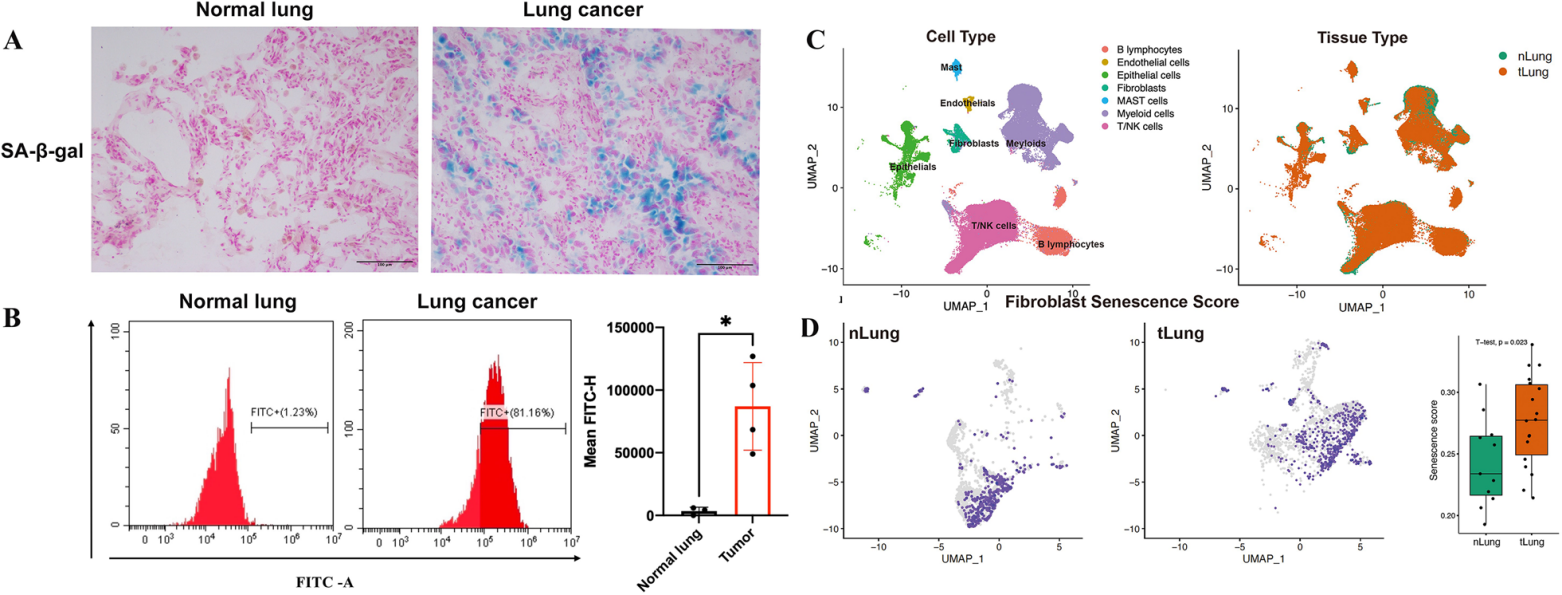
Senescent CAF increased in tumor tissue. **A** Comparison of senescence associated β-galactosidase staining (SA-β-gal staining) was conducted between the tumor tissue and pericancerous tissue. Positive SA-β-gal staining appeared as blue in the tissue. **B** The cell senescence in the tumor tissue and pericarcinoma tissue was analyzed using flow cytometry. **C** UMAP visualization was employed to depict the composition of cell types in lung adenocarcinoma tissue (tLung) and paracancer tissue (nLung). **D** UMAP visualization illustrated the distribution of senescent cancer-associated fibroblasts (CAFs) in lung adenocarcinoma and paracancer tissues

We collected 6 lung adenocarcinoma (LUAD, TNM staging IIIA-IIIB) and normal adjacent tissues in Lung Cancer Center of West China Hospital for SA-β-gal staining to assess tissue senescence (Additional file 2: Table S1). The results revealed the proportion of senescent cells increased in tumor compared to a normal adjacent tissue. (Fig. 1A). Additionally, we collected 4 pairs of lung adenocarcinoma (LUAD, TNM stage IIIA-IIIB) and paracarcinoma samples for measuring fluorescence-labeled β-galactosidase (β-gal) expression using flow cytometry (Additional file 2: Table S1). Consistent with the staining results, a higher percentage of senescent cells was founed in tumor compared to para-cancerous tissues (Fig. 1B).

### The proportion of CAFs in lung adenocarcinoma tissues increased

In order to assess the senescence score of distinct cell subsets in tumor, we integrated two independent single-cell sequencing datasets of lung cancer (GSE123902 and GSE131907), encompassing a total of 28 patients, to calculate senescence score. The findings demonstrated a significantly higher proportion of aged CAFs in lung adenocarcinoma compared to paracancerous tissue (Fig. 1C, D) (P = 0.023).

### Senescent CAFs facilitate the recruitment of neutrophils

**Fig. 2:**
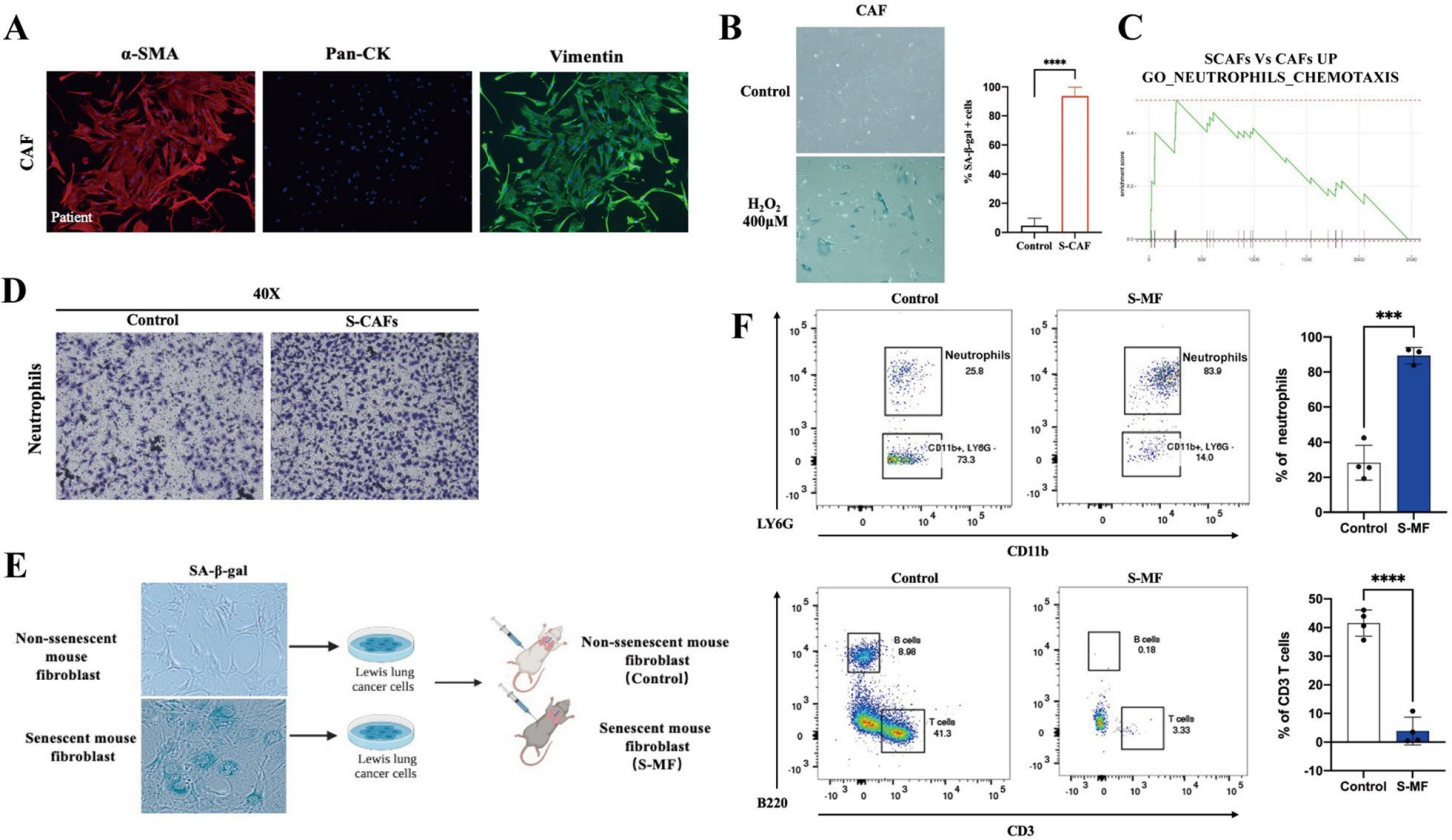
Senescent CAFs in tumor increased neutrophil recruitment and inhibited T cell infiltration. **A** Primary CAFs extracted from lung adenocarcinoma tissue and were identified by cellular immunofluorescence assay (n = 3). α-SMA (red), Vimentin (green), Pan-CK (yellow). **B** H_2_O_2_ was used to construct senescent CAFs in vivo. **C** GSEA of senescent and non-senescent CAFs. **D** Transwell migration assay was used to observe the migration of neutrophils. **E** Flow chart of senescent transplanted tumor model. **F** The proportion of neutrophil and the proportion of T-cell infiltration in the transplanted tumor were detected by flow cytometry

Furthermore, we performed transcriptome sequencing using senescent or non-senescent CAFs. Gene set enrichment analysis (GSEA) revealed that neutrophil chemoattractant pathway was predominantly enriched in senescent CAFs (Fig. 2C). This suggests a possible association between senescent CAFs and enhanced recruitment of neutrophils. To validate these findings, senescent cancer-associated fibroblasts (S-CAFs) were co-cultured with human peripheral blood neutrophils using a Transwell chamber system. Neutrophils were placed in the upper chamber while senescent CAFs were positioned in the lower chamber. The results showed that S-CAFs significantly facilitated neutrophil migration (Fig. 2D). Similarly, we found tumor-associated neutrophil recruitment increased in tumor microenvironment in tumor-transplanted mice (S-MF group), while T lymphocyte infiltration was reduced (Fig. 2F).

## Discussion and conclusion

CAFs represent a crucial mesenchymal constituent in tumor microenvironment. Numerous investigations have documented the ability of CAFs to impede infiltration of immune cells into tumor and exert pro-tumor effects. Consequently, several drugs targeting CAF have been developed. However, their efficacy remains uncertain (NCT04467723) [3]. In recent years, the advent of single-cell sequencing has enabled researchers to further categorize CAFs into distinct subgroups. Our study using scRNA-seq data found senescent CAFs increased in lung adenocarcinoma cohort, which was closely associated with augmented tumor associated neutrophil and diminished T cell. It has been reported neutrophils are capable of releasing reactive oxygen species (ROS) and microRNA-containing particles to induce DNA damage and genetic instability in tumor, thereby accelerating carcinogenesis. Importantly, the release of neutrophil extracellular traps (NETs) under stimulation signals such as IL-8, phorbolol, lipopolysaccharide, etc., formed by citrullination and chromatin deagglutination of histone H3 within the nucleus, contributes to tumor proliferation [4]. Additionally, Alvaro Teijeira's team discovered that NETs interfered with the contact between cytotoxic T cells/NK cells and tumor cells, inhibiting immune cell-mediated killing effects. Removal of NETs from the tumor microenvironment improved the efficacy of immune checkpoint inhibitors [5]. Therefore, we speculate that, the phenomenon in our study that senescent CAFs promoting neutrophil recruitment and thereby inhibiting T cell-induced immunosuppression may also be related to NETs formation; we will further study it in the future.The methods section is elaborated in Additional file 1: Methods.

### Supplementary Information

**Additional file 1:** Methods.

**Additional file 2: Table S1. **Clinicopathological characteristics of patients.

## Data Availability

The datasets used and/or analyzed during the current study are available from the corresponding author upon reasonable request.

## Abbreviations

NSCLC: Non-small cell lung cancer
SASPs: Senescence-associated secreted phenotype
CAFs: Cancer-associated fibroblasts
GSEA: Gene set enrichment analysis
NETs: Neutrophil extracellular traps
S-CAFs: Senescent CAFs
LUAD: Lung adenocarcinoma
scRNA-seq: Single-cell RNA sequencing

## References

[1] Garassino MC, Gadgeel S, Speranza G, Felip E, Esteban E, Dómine M, Hochmair MJ, Powell SF, Bischoff HG, Peled N (2023). Pembrolizumab plus pemetrexed and platinum in nonsquamous non-small-cell lung cancer: 5-year outcomes from the phase 3 KEYNOTE-189 study. J Clin Oncol.

[2] Chibaya L, Snyder J, Ruscetti M (2022). Senescence and the tumor-immune landscape: Implications for cancer immunotherapy. Semin Cancer Biol.

[3] Chen X, Song E (2019). Turning foes to friends: targeting cancer-associated fibroblasts. Nat Rev Drug Discov.

[4] Adrover JM, McDowell SAC, He XY, Quail DF, Egeblad M (2023). NETworking with cancer: the bidirectional interplay between cancer and neutrophil extracellular traps. Cancer Cell.

[5] Cristinziano L, Modestino L, Antonelli A, Marone G, Simon HU, Varricchi G, Galdiero MR (2022). Neutrophil extracellular traps in cancer. Semin Cancer Biol.

