# Supplementary material for: Senescent cancer-associated fibroblasts facilitate tumor associated neutrophil recruitment suppressing tumor immunity
Source: J Transl Med. 2024 Mar 3;22:231. doi: 10.1186/s12967-024-05017-w (PMC10909258; doi:10.1186/s12967-024-05017-w)
Supplement: Supplementary file 1 — Additional file 1: Methods. [file 12967_2024_5017_MOESM1_ESM.docx]

**Methods**

**Sample collection**

A total of 10 patients were included in this study. Inclusion criteria: age > 18 years; diagnosed with pulmonary nodules using chest CT; Choose to undergo surgery. Exclusion criteria: stage IV lung cancer. Clinical and pathological staging was performed according to the 8th edition of the Union for International Cancer Control (UICC) TNM staging system for lung cancer.

**Senescence-associated β-galactosidase staining**

The senescence of the cells was tested on cryosections of lung adenocarcinoma and normal adjacent tissues using senescence-associated β-Galacto-sidase Staining Kit (Beyotime, China) according to the manufacturer’ s instructions. The tissues were washed three times with PBS, fixed with β-galactosidase staining fixative for 15 min at room temperature. Washed again 3 times with PBS, 3 minutes/time, added staining working solution and incubated overnight at 37 C in darkness.

**Single-cell sequence analysis**

We downloaded two independent single-cell sequencing datasets of lung cancer (GSE123902 and GSE131907), encompassing a total of 28 patients. The removal of batch effect was performed. The genetic markers for cellular senescence were acquired from the CellAge database in order to calculate the senescence score of S-CAFs, both in lung adenocarcinoma tissues and paracancerous tissue.

**The extraction of primary cells**

The fresh tissues were rinsed thoroughly and repeatedly three times with PBS buffer containing 5% penicillin and streptomycin. Then it was cutted into 1mm^3 sized tissue blocks, added 10 ml collagenase I and IV and placed in a warm box for digestion for 30min, remove it every 10min and shake well. After thorough digestion, it was transferred into 70 μm mesh screen, centrifuged for 1000rp/m for 5min and incubated at 5% CO2 and 37°C.

**Neutrophils co-culture with tumor cells**

Resuspend neutrophils (5×10^4)^ in 200 ul of serum-free medium and seed them in the upper chamber of a 24-well culture dish; Place the conditioned medium of senescent lung cancer-associated fibroblasts (S-LCAF-CM) and the conditioned medium of non-senescent lung cancer-associated fibroblasts (LCAF-CM) (600μl) into the lower compartment; Incubate at 5% CO2 and 37°C for 48 hours; Wash the plate twice with PBS and fix the neutrophils with 4% paraformaldehyde for 20 minutes. Migrating cells were stained with crystal violet for 20 minutes; Cells that migrated to the lower surface were counted under a microscope (400×) from five random fields. Data are expressed as mean ± SD, and all experiments were repeated independently at least 3 times. Chemotaxis was assessed by cell number.

**The construction of senescent model**

The mouse Lewis lung cancer cell line, mouse fibroblasts (MF), and senescent mouse fibroblasts (S-MF) was cultured and digested with trypsin to make a cell suspension. Mouse Lewis lung cancer cells : MF cells (1:1) were inoculated subcutaneously to establish lung cancer transplant tumors in the control group. Mouse lewis lung cancer cells : S-MF cells (1:1) were inoculated to establish the senescence group.

**Preparation of single-cell suspensions and Immunophenotyping of lymphocytes**

Mice tumor samples were immersed in cold phosphate buffered saline (PBS) immediately after resection. The fresh tissues were washed and minced. Cell suspensions were filtered through a 70 μm strainer, pelleted (500 × g, 5 min, 4 °C), resuspended in 2 ml of 1× red blood cell lysis buffer (Sigma, R7757). The dissociated cells were pelleted again (500 × g, 5 min, 4 °C) and blocked with purified anti-mouse CD16/CD32 mAb 2.4G2 (Mouse BD Fc Block™) for 15 minutes; Adding 100ul PBS containing (CD45, CD3, B220/CD45R, CD11b, LY6G respectively) to each centrifuge tube for staining. Quantiﬁcation of T cells and Neutrophils in two groups was performed by ﬂow cytometry. Finally, the cells were acquired on a FACSCalibur using BD FACS DIVA software.
