# Supplementary material for: Senescent cancer-associated fibroblasts facilitate tumor associated neutrophil recruitment suppressing tumor immunity
Source: J Transl Med. 2024 Mar 3;22:231. doi: 10.1186/s12967-024-05017-w (PMC10909258; doi:10.1186/s12967-024-05017-w)
Supplement: Supplementary file 2 — Additional file 2: Table S1. Clinicopathological characteristics of patients. [file 12967_2024_5017_MOESM2_ESM.docx]

**Additional file 2: Table S1. Clinicopathological characteristics of patients**

| **Characteristics** | | **No** | |
| --- | --- | --- | --- |
| Gender  Female  Male  Age (mean ± SD) | | | 6  4  58.80±6.596 |
| Smoking history | | |  |
|  | Present/ex-smoker | | 3 |
|  | Non-smoker | | 7 |
| Lesion location | | |  |
|  | Right upper lobe | | 4 |
|  | Right middle lobe | | 1 |
|  | Right lower lobe | | 0 |
|  | Left upper lobe | | 5 |
|  | Left lower lobe | | 0 |
| pT stage | | |  |
|  | T1a | | 0 |
|  | T1b | | 0 |
|  | T1c | | 3 |
|  | T2a | | 6 |
|  | T2b | | 0 |
|  | T3 | | 1 |
|  | T4 | | 0 |
| pN stage | | |  |
|  | N0 | | 0 |
|  | N1 | | 0 |
|  | N2 | | 10 |
| pTNM stage | | |  |
|  | IA1 | | 0 |
|  | IA2 | | 0 |
|  | IA3 | | 0 |
|  | IB | | 0 |
|  | IIA | | 0 |
|  | IIB | | 0 |
|  | IIIA | | 9 |
|  | IIIB | | 1 |
|  | IIIC | | 0 |
| Tumor differentiation | | |  |
|  | Well differentiated | | 0 |
|  | Moderately differentiated | | 4 |
|  | Poorly differentiated | | 6 |
|  | NA | | 0 |
